# Neutralization of IL-17 ameliorates uveitis but damages photoreceptors in a murine model of spondyloarthritis

**DOI:** 10.1186/ar3697

**Published:** 2012-01-23

**Authors:** Jelena M Kezic, Tibor T Glant, James T Rosenbaum, Holly L Rosenzweig

**Affiliations:** 1 Casey Eye Institute, Oregon Health & Science University, 3181 SW Sam Jackson Park Rd., Portland, OR 97239, USA; 2 JMK is now affiliated with Centre for Eye Research Australia, University of Melbourne, East Melbourne VIC, 3002 Australia; and Department of Anatomy and Developmental Biology, Monash University, Melbourne, VIC, Australia; 3 Departments of Biochemistry and Orthopedics, Rush University Medical Center, 1735 W. Harrison St., Chicago, IL 60612, USA; 4 Department of Medicine, Oregon Health & Science University, 3181 SW Sam Jackson Park Rd., Portland, OR 97239, USA; 5 Department of Cell and Developmental Biology, Oregon Health & Science University, 3181 SW Sam Jackson Park Rd., Portland, OR 97239, USA; 6 Department of Molecular Microbiology and Immunology, Oregon Health & Science University, 3181 SW Sam Jackson Park Rd., Portland, OR 97239, USA

## Abstract

**Introduction:**

Uveitis, or intraocular inflammatory disease, is a frequent extra-articular manifestation of several forms of arthritis. Despite the frequent co-occurrence of uveitis and arthritis, little is understood of the eye's predisposition to this disease. We recently described a previously unreported uveitis in a murine model of spondyloarthropathy triggered by autoimmunity to aggrecan, a prominent proteoglycan (PG) macromolecule in cartilage. In contrast to the joint and spine, wherein interferon-gamma (IFNγ) deficiency reduced disease, IFNγ deficiency worsened uveitis. Given the regulatory role of IFNγ on the Th17 response and the current focus of anti-interleukin-17 therapeutics in patients with uveitis and spondyloarthritis, we sought to determine the extent to which interleukin (IL)-17 mediates uveitis in the absence of IFNγ.

**Methods:**

Antigen specific T cell cytokine production was measured in splenocyte cultures using multiplex-ELISA. Transgenic (Tg) mice expressing the T cell receptor (TCR) recognizing the dominant arthritogenic epitope in the G1 domain of PG (TCR-Tg), also lacking IFNγ, were immunized with PG. Mice were then systemically administered an anti-IL-17 neutralizing antibody. The onset and severity of peripheral arthritis was evaluated by clinical scoring criteria and histology. Uveitis was assessed using intravital videomicroscopy, which visualizes leukocyte trafficking within the vasculature and tissue of the iris, and by histology.

**Results:**

TCR-Tg splenocytes stimulated *in vitro *with recombinant G1 peptide demonstrated exacerbated production of cytokines, such as macrophage inflammatory protein (MIP)-1α, MIP-1β, IL-1β, and most notably IL-17A as a consequence of IFNγ deficiency. *In vivo*, IL-17 inhibition prevented the component of PG-induced arthritis that occurs independently of IFNγ. Blockade of IL-17 ameliorated the ongoing leukocyte trafficking responses within the iris vasculature and tissue, which coincided with reduced infiltration of leukocytes within the anterior and posterior eye segments. However, the anti-IL-17 treatment resulted in unanticipated photoreceptor toxicity.

**Conclusions:**

These data support a protective, regulatory role for IFNγ in suppression of IL-17-mediated intraocular disease and to a lesser extent, joint disease. The unanticipated photoreceptor toxicity raises some caution regarding the use of anti-IL-17 therapeutics until the mechanism of this potential effect is determined.

## Introduction

Uveitis, or intraocular inflammatory disease, is a leading cause of visual loss and the most common, clinically important extra-articular manifestation in several several diseases such as ankylosing spondylitis (AS), Behçet's disease, and juvenile idiopathic arthritis. Anterior uveitis, wherein the iris tissue is consistently affected, is the most frequently diagnosed type of uveitis in North America and Europe and occurs in as many as 50% of patients with AS [1,2]. Despite the high incidence of uveitis with AS and its closely related spondyloarthropathies [3], the mechanism for their coexistence is not known. Indeed, uveitis has not been a reported feature in mouse models of arthritic disease.

We recently discovered that uveitis develops in a murine model of spondyloarthropathy that arises from autoimmunity to the cartilage proteoglycan (PG) aggrecan [4], which is a proposed potential autoantigen in AS [5]. Experimental PG-induced uveitis appears to replicate to some extent the spectrum of human uveitis that occurs in patients with spondyloarthritis. In this mouse model, disease is induced by immunization of genetically susceptible BALB/c mice with PG or its G1 domain [6]. A progressive and chronic erosive polyarthritis and axial spondylitis ensue [7-9]. PG-arthritis is a T cell-dependent disease [10] in which the Th1 effector response plays an important pathogenic role in the peripheral joint disease [11,12]. Transgenic (Tg) mice expressing the T cell receptor (TCR) recognizing an arthritogenic epitope of PG (denoted here as TCR-Tg mice) develop an earlier onset and more severe form of polyarthritis than wild-type mice [13]. We previously reported an unexpected discordant mechanism of disease in the eyes versus joints with respect to interferon-gamma (IFNγ) in TCR-Tg mice. Mice deficient in IFNγ develop exacerbated uveitis that is characterized by infiltrating granulocytes, whereas the joint and axial disease are ameliorated by IFNγ deficiency [4].

The Th17 signaling axis has emerged as a potential therapeutic target, and anti-IL-17 therapy is currently under evaluation for spondyloarthritis and related diseases, including psoriatic arthritis, psoriasis, inflammatory bowel disease, Behçet's disease and uveitis [14,15]. Given the counter-regulatory role for IFNγ on the Th17 responses, the present study was designed to examine whether the exacerbated uveitis that occurs in the absence of IFNγ results from an imbalance of the Th17 response. Here, we demonstrate that antigen-specific T cell production of IL-17A is exacerbated in the absence of IFNγ. The worsened uveitis that occurs in IFNγ KO mice is inhibited by *in vivo *blockade of IL-17. The remaining arthritis that ensues independently of IFNγ is further reduced as a consequence of IL-17 neutralization. However, our findings reveal unanticipated photoreceptor toxicity subsequent to the treatment with anti-IL-17 antibody, in that the photoreceptors are frequently obliterated. We conclude that IFNγ exerts a critical regulatory role in suppression of IL-17, which is pathogenic in uveitis. Our observations provide insight into a relatively common but poorly understood eye disease and could have potential dual clinical relevance as to how we approach anti-IL-17 therapeutics in patients with uveitis and spondyloarthritis.

## Materials and methods

### Mice, immunizations and treatment with anti-IL-17 antibody

TCR-Tg mice [13] that were backcrossed 10 generations onto a BALB/c background were inter-crossed with IFNγ KO mice on BALB/c background (The Jackson Laboratory, Bar Harbor, ME, USA); hereon abbreviated GKO/TCR-Tg mice. Housing and experimentation on mice was carried out under approval of the Oregon Health and Science University Animal Care and Use Guidelines. Female, 16-week-old mice were immunized intraperitoneally (i.p.) with 100 μg purified human cartilage PG in 200 μl phosphate-buffered saline (PBS, pH 7.4) emulsified with dimethyl dioctadecyl ammonium bromide (DDA; Sigma-Aldrich, St. Louis, MO, USA)), along with 1.5 μg pertussis toxin (Sigma). Mice received an additional i.p. injection of pertussis toxin 48 h later, as previously described [4,16]. Control groups received PBS/DDA and pertussis toxin injections without PG antigen. For neutralization of IL-17, mice were administered an i.p. injection of 100 μg of anti-IL-17 (anti-IL-17A and IL-17A/F heterodimer; monoclonal rat IgG2a, clone # 50104) blocking antibody or 100 μg of rat IgG2a (isotype control antibody, IC; R&D Systems, Minneapolis, MN, USA)) at the time of PG-immunization and weekly thereafter. Mice were assessed weekly for clinical manifestations of peripheral arthritis in a masked fashion using an established scoring method as previously described [4,16]. The scores ranged from 0 to 4 per paw based on swelling, redness, deformities and ankylosis. The data are represented as the cumulative score for all four paws, resulting in a maximum of 16 per mouse.

### Intravital videomicroscopy

Intraocular inflammation of the iris vasculature and tissue in mice anesthetized with 1.7% isoflurane was video recorded as previously described [4,17,18]. Digital videos (10 sec each) of three independent regions of the iris were captured with a black and white video camera (Kappa Scientific, Gleichen, Germany) on an epifluorescence intravital microscope (modified Orthoplan; Leica, Wetzlar, Germany). Diameter and length of each vessel segment or iris tissue as well as leukocyte phenotype (that is, rolling, adhering, infiltrating) were quantified off-line using Image J analysis software available from NIH.

### Histopathology and uveitis scoring

Eyes and decalcified knee joints were fixed and prepared for paraffin embedding, and 7 μm tissue sections were stained with hematoxylin and eosin (H&E) as previously reported [19,20]. For uveitis assessment, eyes were sectioned through the pupillary-optic nerve axis at four different depths and three sections from each depth were examined by a masked observer. Leukocyte infiltration into the aqueous humor of the anterior segment and vitreous body of the posterior segment was quantified as the mean number of cells per section per eye. Structural, morphological and photoreceptor changes to the retina were also assessed using an ocular histopathology grading system, which is indicated as the "structural score" [4]. Both eyes in each mouse were assessed.

### Antigen-specific *in vitro *splenocyte stimulation

Splenocytes collected from three individual TCR-Tg or GKO/TCR-Tg mice were plated (2 × 10^6 ^cells/ml) in 96-well plates (BD Biosciences, San Diego, CA, USA) as described [21], and cells were stimulated in quadruplicate wells with 20 μg/ml of purified recombinant human (rhG1) domain of aggrecan. Cytokines were measured in supernatants 48 h post stimulation with Luminex multiplex ELISA (Millipore, Bedford, MA, USA). Data were analyzed using BeadView Multiplex Analysis Software (version 1.0, Upstate Cell Signaling Solutions, Lake Placid, NY, USA)).

### Statistical analysis

Data are represented as mean ± SEM. Experiments were reproduced at least twice. Means were compared and statistical differences were determined with two-way and one-way analysis of variance (ANOVA) with two-tailed, Students *t*-test *post hoc *analyses. For the nonparametric clinical arthritis scores and ocular histopathology scores, the Mann-Whitney U test was used (Prism; Graph Pad Software, La Jolla, CA, USA). Probability values < 0.05 were considered statistically significant.

## Results

### IFNγ is an essential protective factor against uveitis triggered by proteoglycan autoimmunity

**Figure 1:**
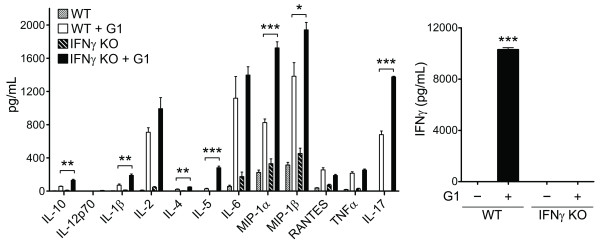
**IFNγ-deficiency results in an altered cytokine profile including exacerbated IL-17A production**. Antigen specific T cell cytokine production was measured in splenocyte cultures from TCR-Tg mice with intact IFNγ (WT) or TCR-Tg mice lacking IFNγ (IFNγ KO) that were stimulated with the arthritogenic G1 peptide carrying the TCR-specific epitiope. Cytokine levels in the supernatants were quantified by multiplex-ELISA (Luminex) at 48 h post stimulation. Data are the mean ± SEM of values combined from three independent experiments. * *P *< 0.05, ** *P *< 0.001, *** *P *< 0.0001 comparison between genotypes of G1-stimulated cells.

As previously described [4], TCR-Tg mice lacking IFNγ (hereon abbreviated GKO/TCR-Tg) develop exacerbated uveitis, which is characterized by increased intravascular cell trafficking responses within the iris, increased glial activation and infiltration of granulocytes. We initially examined the effects of IFNγ deficiency on cytokine production *in vitro *in response to T cell stimulation with the arthritogenic G1 peptide of PG (Figure 1). Enhanced production of cytokines, such as the Th2-related eosinophilic cytokine, IL-5, as well as higher production of neutrophil chemokines (macrophage inflammatory protein (MIP)-1α and MIP-1β) occurred as a consequence of IFNγ-deficiency. This observation is consistent with the histopathology and predominance of neutrophils and eosinophils observed in uveitic eyes as previously reported in mice lacking IFNγ [4]. IL-1β and most notably IL-17A production were markedly enhanced, suggestive of a heightened Th17 response in the absence of IFNγ. Negligible amounts of IL-23p40 or IL-23p19 were detected (data not shown). The normally abundant IFNγ production (plotted on a separate axis, right panel) was abolished in GKO/TCR-Tg mice as expected. These data indicate that in the absence of endogenous IFNγ, IL-17 production is enhanced that could in turn mediate uveitis.

### PG-induced uveitis is dependent on IL-17 in the absence of IFNγ

**Figure 2:**
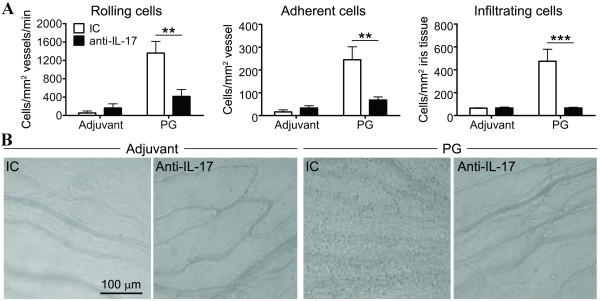
**Cellular trafficking responses exacerbated in the iris by IFNγ deficiency areabrogated by IL-17A blockade**. (**A**) Weekly i.p. injections of anti-IL-17A blocking antibody or isotype-matched control antibody (IC) were administered into PG-immunized GKO/TCR-Tg mice. Adjuvant-immunized control mice were also administered the anti-IL-17A blocking antibody and IC. The leukocyte trafficking response within the iris was assessed by intravital microscopy at three weeks following PG-immunization. ** *P *< 0.001, *** *P *< 0.0001 comparison between treatments: anti-IL-17 and IC antibodies (*n *= 8 mice/adjuvant groups and *n *= 12 mice/PG-immunized groups). (**B**) Representative images captured by intravital videomicroscopy demonstrate the increased leukocyte infiltration within the iris tissue of PG-immunized, IC control mice, which is completely blocked by anti-IL-17A treatment.

Given the emerging data supporting a key role for IL-17 in patients with AS and related spondyloarthropathies [22,23], we tested whether systemically administered blocking antibody to IL-17 would alter the onset and/or severity of uveitis occurring in the absence of IFNγ. Using intravital videomicroscopy, a technique that allows us to visualize ongoing cellular trafficking responses *in vivo *within the iris vasculature and tissue, we examined ocular inflammation at three weeks post immunization, which is a time previously established to consistently coincide with uveitis in this model [4]. As expected, mice that were immunized with adjuvant alone showed negligible ocular inflammation regardless of the antibody treatment (Figure 2A). In contrast, PG immunization of mice treated with the isotype-matched control antibody (IC) resulted in increased leukocyte rolling and adherence within the microvasculature of the iris (Figure 2A) and subsequent infiltration within the iris tissue itself (as depicted in images captured by intravital microscopy (Figure 2B). Neutralization of IL-17 in the PG-immunized mice had a profound impact on the cellular trafficking response, as all cellular responses (rolling, adherence and infiltration) in the iris were abolished.

**Figure 3:**
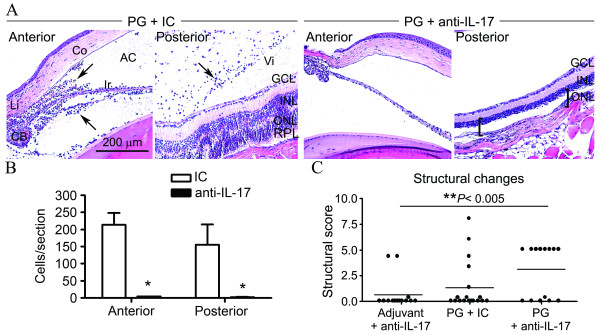
**IL-17 inhibition prevents uveitis but results in photoreceptor toxicity**. (**A**) PG-immunized GKO/TCR-Tg mice were administered weekly i.p. injections of anti-IL-17A blocking antibody or isotype-matched control antibody (IC). Control mice were immunized with adjuvant and the anti-IL-17A blocking antibody or IC. Histological assessment of uveitis was performed at three weeks post PG-immunization. Representative images of the anterior and posterior eye segments are shown. Increased leukocyte infiltration in IC control mice is indicated by arrows (two left panels) (H&E stain). The brackets on the far right panel (anti-IL-17 treated group) indicate the space where the ONL and photoreceptor layers would normally be present, but which are absent. Original magnification: 200X. AC, anterior chamber; Co, cornea; CB, ciliary body, Ir, iris; Li, limbus; Vi, vitreous, GCL, ganglion cell layer, INL, inner nuclear layer, ONL, outer nuclear layer, PRL, photoreceptor layer. (**B**) The infiltrating cells present within the aqueous humor of the anterior eye segment or vitreous body of the posterior eye segment were quantified. (**C**) Structural changes within the retina were scored in both eyes of each mouse. Data points represent individual eyes from each mouse. * *P *< 0.05 comparison between treatments: anti-IL-17A and IC antibodies (*n *= 7 mice/adjuvant groups; *n *= 9 mice/PG-immunized groups).

Histopathological examination revealed a near-complete absence of inflammation in both the anterior and posterior eye segments as a consequence of anti-IL-17 therapy (Figure 3). In PG-immunized mice treated with the isotype control antibody an increased presence of infiltrated leukocytes within the aqueous humor, iris, ciliary body, choroid and vitreous body is visualized (as depicted in representative images Figure 3A). Consistent with our prior report, retinal damage such as folding of the inner nuclear layer (INL) and outer nuclear layer (ONL) layers is also a pathological feature of uveitis in mice lacking IFNγ (Figure 3A), which is graded using a structural scoring system [4]. In contrast, blockade of IL-17 significantly diminished the number of cells in the aqueous humor of the anterior segment as well as the cells in the vitreous body of the posterior segment (Figure 3A, B). Likewise, IL-17 blockade abolished the retinal vasculitis and retinal folding found in several IFNγ-deficient mice with PG-induced uveitis (Figure 3C). Despite the abolishment of inflammatory changes in the posterior segment upon IL-17 inhibition, a near-complete ablation of the ONL and photoreceptor layer (PRL) was observed in 9 of the 16 eyes examined (indicated by brackets in Figure 3A). This damage would be expected to result in complete vision loss. As indicated by the brackets, there is a space where the ONL and photoreceptor layers would normally be present, but which are instead missing in PG-immunized mice treated with anti-IL-17A antibody. The depletion in these layers could be a consequence of prior toxicity or cellular death, which may be attributed to these mice having a higher disease structural score then most of the mice that had received isotype-matched control antibody (Figure 3C). We also observed two eyes with similar retinal toxicity in adjuvant-immunized mice that were treated with anti-IL-17 antibody as indicated in the clinical scores for structural changes (Figure 3C). These data support a pathogenic role for IL-17 in uveitis; but PG-induced inflammation also increases the susceptibility of the eyes to the anti-IL-17-induced retinal toxicity.

### IL-17 mediates arthritis in the absence of IFNγ

**Figure 4:**
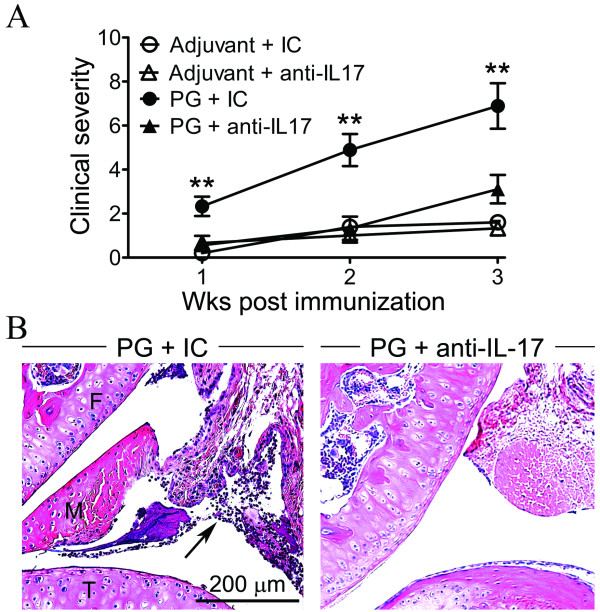
**IFNγ regulates the requirement for IL-17 in PG-induced arthritis**. PG-immunized GKO/TCR-Tg mice were administered weekly i.p. injections of anti-IL-17A blocking antibody or isotype-matched control antibody (IC). Mice were also immunized with adjuvant along with anti-IL-17A blocking antibody and IC. The peripheral arthritis was assessed as a function of time. (**A**) Clinical arthritic scores in GKO/TCR-Tg mice treated with anti-IL-17A or IC; ** *P *< 0.001 comparison between anti-IL-17A and IC antibody treatments in PG-immunized mice (*n *= 8 mice/adjuvant groups and *n *= 12 mice/PG-immunized groups). (**B**) Representative histological images of arthritic knee joints of GKO/TCR-Tg mice treated with IC or anti-IL-17 at three weeks post immunization (F, femur; T, tibia).

PG-induced peripheral arthritis is considered to be primarily a Th1 dependent disease that occurs independently of IL-17 [11,24,25]. We have confirmed the pathogenic role for IFNγ in promoting peripheral arthritis as well as axial disease in the TCR-Tg mice [4]. However, there is a precedent for IFNγ regulating the dependency of IL-17 in arthritis [26], and we demonstrate here that the arthritis ensuing in the absence of IFNγ in TCR-Tg mice is mediated by IL-17. PG-induced arthritis that was already attenuated by the absence of IFNγ was effectively prevented by anti-IL-17 (Figure 4A). The protective effects of IL-17 blockade observed clinically were corroborated by histological changes (Figure 4B), wherein the synovial inflammation and infiltrated leukocytes were reduced. These data support a combinatorial role for Th1 and Th17 responses in promoting arthritis while IFNγ suppresses uveitis through an IL-17-dependent mechanism.

## Discussion

Our prior data demonstrated an important protective role for IFNγ in uveitis in a murine model of progressive spondyloarthritis. Here, we tested the extent to which IL-17 mediates uveitis occurring in the absence of IFNγ. Antigen-specific T cell production of IL-17A was exacerbated *in vitro *as a consequence of IFNγ deficiency. Assessment of leukocyte trafficking within the iris microvasculature and tissue by intravital videomicroscopy in mice treated with anti-IL-17 neutralizating antibody revealed impaired intravascular responses, such as rolling and adherence that coincided with fewer infiltrating cells in the iris tissue. The histopathology demonstrated that IL-17 blockade significantly reduced the number of infiltrating leukocytes within the aqueous humor and vitreous body. The peripheral arthritis that accompanies PG-induced uveitis was also diminished in mice that received the anti-IL-17 blocking antibody. However, despite protective effects of IL-17 inhibition on PG-induced uveitis, unexpected retinal toxicity was observed. Collectively, our data support an important role for IL-17 in the pathogenesis of PG-induced uveitis, which is negatively regulated by IFNγ.

Uveitis encompasses a heterogeneous group of diseases, and even though it can arise from microbial or viral infection, it is frequently diagnosed in inflammatory diseases involving the joints, such as ankylosing spondylitis, juvenile idiopathic arthritis, Behçet's disease and many others. The underlying mechanisms that result in the co-existence of uveitis and arthritis/spondylitis are poorly understood. An important association exists between HLA-B27 and anterior uveitis in patients with ankylosing spondylitis. Approximately 90% of affected individuals have the inherited B27 allele [27], but HLA-B27 does not account for all of the genetic risk for disease, nor the extent to which it influences the onset and severity of uveitis versus spondyloarthritis. For example, HLA-B27 transgenic rats spontaneously develop spondyloarthropathy but do not develop uveitis [28,29]. This underscores the complexity of multisystem, immune-mediated polygenic diseases, in which environmental factors and/or endogenous microflora can contribute to the pathogenesis of disease within the different target organs.

Although it is not known whether the pathogenic mechanisms in uveitis are the same as arthritis and/or spondylitis, we initially hypothesized that the underlying basic mechanisms would be shared between these two organs in mice immunized with PG. Somewhat paradoxically, however, we found that IFNγ deficiency reduced disease in the joint and spine but worsened disease in the eye [4]. The discordant effects of IFNγ underscore the complexity of how immune-mediated diseases target multiple organs differentially. This phenomenon has presented considerable challenges in the treatment of uveitis in patients with multisystem diseases, such as Behçet's disease or AS. For example, the TNFα inhibitor, etanercept, diminishes arthritic symptoms in a subgroup of patients with AS [30,31], but it does not always have comparable beneficial effects for uveitis in the same patients [32]; or it can even lead to increased severity of uveitis [33]. Understanding the unique aspects of individually affected tissues is clearly pertinent to the design of optimal therapies.

The data presented here suggest that IFNγ is protective in the eye by virtue of being counter-regulatory to the IL-17 response. Studies with various KO mice or blocking antibodies have dissected the separate roles of Th1 and Th17 effector responses in the peripheral arthritis aspect of the PG-induced disease model [11,24,25], wherein IFNγ serves an an important regulatory role in arthritis [26]. These studies indicate a dominant pathogenic role for IFNγ and the Th1 effector response over that of the Th17 response in arthritis. We recently demonstrated a similar pathogenic role for IFNγ in peripheral arthritis and extended its contribution to the axial disease that develops in TCR-Tg mice [4]. Our findings would indicated that although the Th17 response is not considered critical in the development of PG-induced joint disease, it does appear to be a prevailing response contributing to uveitis.

Th17 cells have been reported to play a role in induction of the animal model of posterior uveitis, experimental autoimmune uveitis (EAU) [34] and have been implicated in uveitis clinically [35,36]. IL-17 is thought to contribute to disease through its ability to promote recruitment and activation of neutrophils, which is consistent with our previously reported increase in granulocyte numbers in PG-immunized mice lacking IFNγ [4]. The production of IL-17 from T cells is well-described, yet additional cellular sources including γδ T cells, NKT cells, macrophages, mast cells and neutrophils may be just as important as the T cells [37-40]. Indeed, a recent report supports the role of innate immune cells rather than T cells in expression of IL-17 in the joints of patients with spondyloarthritis [41]. Thus, the pathology of uveitis observed in response to PG-immunization could involve coordinated events between the T cells of the Th1 and Th17 lineages and innate immune cells, which then propagate tissue damage within uveitic eyes. Future studies that compare the cytokine sources along with their targets within uveitic eyes versus the arthritic joints will be informative.

The IL-23/IL-17 axis is a major focus of spondyloarthritis research, and many pharmaceutical companies are actively working on strategies to block IL-17 in uveitis, AS and related spondyloarthropathies [14,15,36,42]. Therefore, it is critical to understand how this cytokine can contribute to immune-mediated uveitis models in mice, and possibly in humans. Although reduced clinical signs of arthritis and ocular disease following anti-IL-17 treatment suggest great therapeutic potential for the use of this antibody, our observations of retinal toxicity indicate the need to proceed cautiously. We were unable to measure visual acuity, however the observed depletion of photoreceptors and ONL would be expected to result in near to complete vision loss. This observation brings to question why inhibition of IL-17 may result in toxicity within the eye. We are not aware of any reports that demonstrate a role or involvement of IL-17A in the survival of retinal cells. Nonetheless, a number of studies implicate IL-17A involvement in apoptotic or cell survival responses in different cell types and diseases [43-45], suggesting its potential role in cellular viability within the eye. Retinal toxicity derived from anti-IL-17 antibody treatments has not been previously reported in other murine uveitis models [34,46,47], indicating that the toxicity could possibly involve a combination of events within this particular disease model, such as glial cell activation within the retina, the presence of PG-antigen within the retina, and/or ongoing systemic disease [4]. At the time examined here (three weeks post PG-immunization), the photoreceptor toxicity did not coincide with increased expression of GFAP (glial fibrillary acidic protein, a marker of glial activation, data not shown). However, we cannot rule out the contribution of glial activation at times preceding photoreceptor toxicity. Studies are underway to elucidate whether inhibiting the Th17 response through targeting IL-17R or the upstream cytokine IL-23 reduces uveitis without photoreceptor damage. These studies would inform us to the potential toxicity effects of this particular antibody versus the contribution of the Th17 pathway in photoreceptor viability. The observations presented here yield valuable information regarding contrasting mechanisms of disease within different portions of the body and could have an impact on how we approach therapy in complex, inflammatory diseases.

## Conclusions

The present study demonstrates an important regulatory role for IFNγ in uveitis associated with a murine model of spondyloarthropathy. Antigen-specific activation of naive TCR-Tg T cells lacking IFNγ in culture resulted in enhanced cytokine levels of IL-17A, which correlated with worsened uveitis but not arthritis in PG-immunized mice. Blockade of IL-17 *in vivo *ameliorated the ongoing cell trafficking responses within the iris vasculature and iris tissue, which coincided with diminished leukocyte accumulation within the aqueous humor and vitreous body. The residual arthritis ensuing in the absence of IFNγ was further reduced by anti-IL-17 neutralization. These data support a combinatorial role for Th1 and Th17 responses in arthritis, which is in opposition to the eye wherein we discovered a counter-regulatory role for Th1 and Th17 responses in uveitis. Despite the beneficial anti-inflammatory effects of the anti-IL-17 blocking antibody, caution must be exercised in developing anti-IL-17 therapeutics for uveitis due to the unexpected photoreceptor toxicity. Investigation of simultaneous eye and joint disease within a mouse could reveal important insights into how we approach therapy in uveitic patients with inflammatory diseases such as AS.

## Abbreviations

AC: anterior chamber; AS: ankylosing spondylitis; CB: ciliary body; Co: cornea; DDA: dimethyl dioctadecyl ammonium bromide; F: femur; GCL: ganglion cell layer; GKO/TCR-Tg mice: TCR-Tg mice deficient in IFNγ; IC: isotype control antibody; INL: inner nuclear layer; i.p.: intraperitoneal; Ir: iris; Li: limbus; ONL: outer nuclear layer; PG: the proteoglycan aggrecan; PRL: photoreceptor layer; T: tibia; TCR-Tg mice: transgenic mice expressing the TCR for the arthritogenic epitope of PG; Vi: vitreous; WT: wild-type.

## Competing interests

The authors declare they have no competing interests except for JTR, who has consulted for Novartis, Amgen, Genentech, Pfizer, Abbott, UBC, Xoma and Allergan.

## Authors' contributions

JK carried out the experiments, participated in acquisition of the data, analysis and interpretation of the data, and the manuscript preparation. TG participated in the acquisition of *in vitro *data through provision of recombinant G1 domain, interpretation of data and helped to draft the manuscript. JR participated in the design of the study, interpretation of data, and manuscript preparation. HR conceived of the study, participated in the design, data analysis, interpretation and manuscript preparation. All authors read and approved the final manuscript.
